# (2-Carb­oxy­acetato-κ^2^ *O* ^1^,*O* ^1′^)(*rac*-5,5,7,12,12,14-hexa­methyl-1,4,8,11-tetra­aza­cyclo­tetra­decane-κ^4^ *N*,*N*′,*N*′′,*N*′′′)nickel(II) perchlorate acetonitrile solvate

**DOI:** 10.1107/S1600536810042637

**Published:** 2010-10-30

**Authors:** Guang-Chuan Ou, Seik Weng Ng

**Affiliations:** a Department of Biology and Chemistry, Hunan University of Science and Engineering, Yongzhou Hunan 425100, People’s Republic of China; b Department of Chemistry, University of Malaya, 50603 Kuala Lumpur, Malaysia

## Abstract

In the crystal structure of the title salt, [Ni(C_3_H_3_O_4_)(C_16_H_36_N_4_)]ClO_4_·CH_3_CN, the macrocycle folds around the Ni^II^ atom, which is also chelated by the carboxyl­ate monoanion. The geometry is a distorted NiN_4_O_2_ octa­hedron. The formula units are connected by N—H⋯O hydrogen bonds into centrosymmetric dimers. Further N—H⋯O and O—H⋯O hydrogen bonds link the complex mol­ecules and the perchlorate ions.

## Related literature

For three related structures, see: Jiang *et al.* (2005 ▶); Ou, Zhang & Yuan (2009 ▶); Ou, Zhou & Ng (2009 ▶).
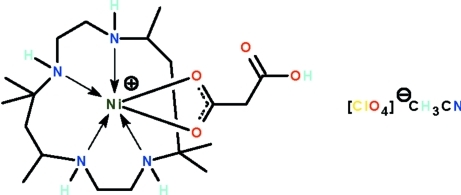


## Experimental

### 

#### Crystal data

[Ni(C_3_H_3_O_4_)(C_16_H_36_N_4_)]ClO_4_·C_2_H_3_N
*M* *_r_* = 586.76
Triclinic, 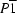

*a* = 9.5236 (4) Å
*b* = 10.1766 (4) Å
*c* = 15.3372 (6) Å
α = 92.899 (1)°
β = 107.388 (1)°
γ = 106.516 (1)°
*V* = 1344.99 (9) Å^3^
*Z* = 2
Mo *K*α radiation
μ = 0.87 mm^−1^
*T* = 173 K
0.45 × 0.40 × 0.20 mm

#### Data collection

Bruker SMART APEX diffractometer
Absorption correction: multi-scan (*SADABS*; Sheldrick, 1996 ▶) *T*_min_ = 0.695, *T*_max_ = 0.845
11181 measured reflections
5701 independent reflections
4946 reflections with *I* > 2σ(*I*)
*R*_int_ = 0.019

#### Refinement

*R*[*F* ^2^ > 2σ(*F* ^2^)] = 0.031
*wR*(*F* ^2^) = 0.090
*S* = 1.03
5701 reflections
330 parameters
1 restraint
H atoms treated by a mixture of independent and constrained refinement
Δρ_max_ = 0.55 e Å^−3^
Δρ_min_ = −0.54 e Å^−3^

### 

Data collection: *SMART* (Bruker, 2003 ▶); cell refinement: *SAINT* (Bruker, 2003 ▶); data reduction: *SAINT*; program(s) used to solve structure: *SHELXS97* (Sheldrick, 2008 ▶); program(s) used to refine structure: *SHELXL97* (Sheldrick, 2008 ▶); molecular graphics: *X-SEED* (Barbour, 2001 ▶); software used to prepare material for publication: *publCIF* (Westrip, 2010 ▶).

## Supplementary Material

Crystal structure: contains datablocks global, I. DOI: 10.1107/S1600536810042637/bt5383sup1.cif

Structure factors: contains datablocks I. DOI: 10.1107/S1600536810042637/bt5383Isup2.hkl

Additional supplementary materials:  crystallographic information; 3D view; checkCIF report

## Figures and Tables

**Table 1:** Hydrogen-bond geometry (Å, °)

*D*—H⋯*A*	*D*—H	H⋯*A*	*D*⋯*A*	*D*—H⋯*A*
O3—H3o⋯O1^i^	0.83 (1)	1.84 (1)	2.669 (2)	173 (3)
N1—H1⋯O4^i^	0.88	2.19	3.038 (2)	161
N2—H2⋯O5	0.88	2.21	3.052 (2)	160
N3—H3⋯O6^ii^	0.88	2.44	3.291 (2)	163
N4—H4⋯O7	0.88	2.24	3.080 (2)	161

Symmetry codes: (i) 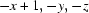; (ii) 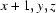.

**Fig. 1.**
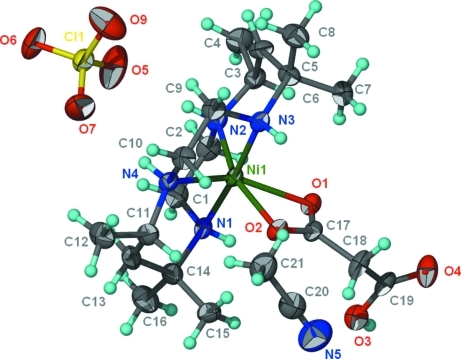
Thermal ellipsoid plot (Barbour, 2001) of [Ni(C16H36N4)(C3H3O4)]+.ClO4-.CH3CN at the 70% probability level; hydrogen atoms are shown as spheres of arbitrary radius.

## supplementary crystallographic information

### Comment

We have reported adducts of 5,5,7,12,12,14-hexamethyl-1,4,8,11-tetraazacyclotetradecane with nickel carboxylates; in the synthesis, the perchlorate counterion reactant is sometimes incorporated into the crystal structure, so that the macrocycle-chelated entity is formally a mono-cation. When a dicarboxylic acid is used, only one carboxylic acid –CO_2_H end is deprotonated, as noted in the butenoate (Jiang *et al.*, 2005), phthalate (Ou,  Zhang & Yuan, 2009) and malate (Ou,   Zhou    & Ng, 2009) salts. In Ni(C_16_H_36_N_4_)(C_3_H_3_O_4_)^+.^ClO_4_^-.^CH_3_CN (Scheme I), the macrocycle folds around the nickel(II) atom, which is also chelated by the carboxylate monoanion. The geometry is an NiN_4_O_2_ octahedron (Fig. 1). Adjacent cations and anions are linked by N–H···O hydrogen bonds to form a centrosymmetric dimer (Table 1). The acetonitrile molecule does not engage in any interaction.

### Experimental

Malonic acid (0.208 g, 2 mmol) and sodium hydroxide (0.08 g, 2 mmol) were dissolved in water (10 ml). To the solution was added [Ni(*rac*-*L*)](ClO_4_)_2_ (0.108 g, 2 mmol) dissolved in acetonitrile (10 ml) (*rac*-*L* = 5,5,7,12,12,14-hexamethyl-1,4,8,11tetraazacyclotetradecane). The solution was left to stand at room temperature; blue crystals formed after several days.

### Refinement

Carbon-bound H-atoms were placed in calculated positions (C–H 0.95–1.00 Å) and were included in the refinement in the riding model approximation, with *U*_iso_(H) set to 1.2–1.5*U*_eq_(C).

The amino H-atoms were similarly restrained [N–H 0.88 Å]  with *U*_iso_(H) set to 1.2–1.5*U*_eq_(N).

The carboxylic acid H-atom was located in a difference Fourier map, and was refined isotropically with a distance restraint of O–H 0.84±0.01 Å.

### Crystal data

**Table:**

[Ni(C_3_H_3_O_4_)(C_16_H_36_N_4_)]ClO_4_·C_2_H_3_N	*Z* = 2
*M**_r_* = 586.76	*F*(000) = 624
Triclinic, *P*1	*D*_x_ = 1.449 Mg m^−^^3^
Hall symbol: -P 1	Mo *K*α radiation, λ = 0.71073 Å
*a* = 9.5236 (4) Å	Cell parameters from 7463 reflections
*b* = 10.1766 (4) Å	θ = 2.3–27.0°
*c* = 15.3372 (6) Å	µ = 0.87 mm^−^^1^
α = 92.899 (1)°	*T* = 173 K
β = 107.388 (1)°	Block, blue
γ = 106.516 (1)°	0.45 × 0.40 × 0.20 mm
*V* = 1344.99 (9) Å^3^	

### Data collection

**Table:**

Bruker SMART APEX diffractometer	5701 independent reflections
Radiation source: fine-focus sealed tube	4946 reflections with *I* > 2σ(*I*)
graphite	*R*_int_ = 0.019
φ and ω scans	θ_max_ = 27.0°, θ_min_ = 1.4°
Absorption correction: multi-scan (*SADABS*; Sheldrick, 1996)	*h* = −12→12
*T*_min_ = 0.695, *T*_max_ = 0.845	*k* = −12→12
11181 measured reflections	*l* = −19→19

### Refinement

**Table:**

Refinement on *F*^2^	Primary atom site location: structure-invariant direct methods
Least-squares matrix: full	Secondary atom site location: difference Fourier map
*R*[*F*^2^ > 2σ(*F*^2^)] = 0.031	Hydrogen site location: inferred from neighbouring sites
*wR*(*F*^2^) = 0.090	H atoms treated by a mixture of independent and constrained refinement
*S* = 1.03	*w* = 1/[σ^2^(*F*_o_^2^) + (0.0459*P*)^2^ + 0.9446*P*] where *P* = (*F*_o_^2^ + 2*F*_c_^2^)/3
5701 reflections	(Δ/σ)_max_ = 0.001
330 parameters	Δρ_max_ = 0.55 e Å^−^^3^
1 restraint	Δρ_min_ = −0.54 e Å^−^^3^

### Fractional atomic coordinates and isotropic or equivalent isotropic displacement parameters (Å^2^)

**Table:**

	*x*	*y*	*z*	*U*_iso_*/*U*_eq_	
Ni1	0.44427 (3)	0.26403 (2)	0.226567 (15)	0.01746 (8)	
Cl1	0.11013 (6)	0.48419 (5)	0.33697 (4)	0.02939 (12)	
O1	0.53455 (15)	0.20193 (14)	0.11561 (9)	0.0226 (3)	
O2	0.63737 (15)	0.19233 (14)	0.26257 (9)	0.0232 (3)	
O3	0.60045 (17)	−0.08012 (15)	0.05987 (10)	0.0296 (3)	
H3O	0.565 (3)	−0.119 (3)	0.0053 (9)	0.048 (8)*	
O4	0.75796 (18)	0.07704 (17)	0.00664 (10)	0.0366 (4)	
O5	0.0676 (2)	0.4372 (3)	0.24039 (14)	0.0642 (6)	
O6	−0.0232 (2)	0.4920 (2)	0.35800 (12)	0.0452 (4)	
O7	0.1767 (2)	0.3921 (2)	0.39064 (17)	0.0605 (6)	
O9	0.2239 (2)	0.6171 (2)	0.35917 (14)	0.0593 (5)	
N1	0.26807 (18)	0.07075 (16)	0.17735 (11)	0.0211 (3)	
H1	0.2850	0.0349	0.1295	0.025*	
N2	0.27765 (18)	0.34713 (17)	0.15032 (11)	0.0225 (3)	
H2	0.2373	0.3773	0.1888	0.027*	
N3	0.60664 (18)	0.46469 (16)	0.27909 (11)	0.0211 (3)	
H3	0.6984	0.4527	0.2954	0.025*	
N4	0.41760 (19)	0.26668 (16)	0.35641 (11)	0.0212 (3)	
H4	0.3344	0.2903	0.3533	0.025*	
N5	0.9236 (3)	0.0264 (3)	0.37545 (18)	0.0560 (6)	
C1	0.1245 (2)	0.1067 (2)	0.13879 (15)	0.0292 (4)	
H1A	0.0876	0.1312	0.1894	0.035*	
H1B	0.0427	0.0260	0.0967	0.035*	
C2	0.1553 (2)	0.2276 (2)	0.08669 (14)	0.0282 (4)	
H2A	0.1890	0.2023	0.0349	0.034*	
H2B	0.0593	0.2523	0.0609	0.034*	
C3	0.3327 (2)	0.4644 (2)	0.10165 (13)	0.0255 (4)	
H3A	0.3813	0.4323	0.0588	0.031*	
C4	0.1996 (3)	0.5125 (3)	0.04463 (16)	0.0371 (5)	
H4A	0.1210	0.4342	0.0002	0.056*	
H4B	0.2396	0.5861	0.0113	0.056*	
H4C	0.1532	0.5480	0.0858	0.056*	
C5	0.4547 (2)	0.5866 (2)	0.17060 (14)	0.0276 (4)	
H5A	0.4677	0.6684	0.1380	0.033*	
H5B	0.4118	0.6060	0.2194	0.033*	
C6	0.6159 (2)	0.5764 (2)	0.21848 (13)	0.0249 (4)	
C7	0.6890 (2)	0.5397 (2)	0.14827 (14)	0.0291 (4)	
H7A	0.6248	0.4492	0.1117	0.044*	
H7B	0.7931	0.5362	0.1809	0.044*	
H7C	0.6959	0.6104	0.1072	0.044*	
C8	0.7192 (3)	0.7183 (2)	0.27434 (16)	0.0346 (5)	
H8A	0.8235	0.7139	0.3057	0.052*	
H8B	0.6752	0.7435	0.3204	0.052*	
H8C	0.7251	0.7880	0.2327	0.052*	
C9	0.5823 (2)	0.5038 (2)	0.36648 (13)	0.0269 (4)	
H9A	0.4917	0.5381	0.3531	0.032*	
H9B	0.6745	0.5791	0.4063	0.032*	
C10	0.5551 (2)	0.3792 (2)	0.41571 (13)	0.0268 (4)	
H10A	0.6472	0.3470	0.4308	0.032*	
H10B	0.5389	0.4049	0.4742	0.032*	
C11	0.4025 (2)	0.1328 (2)	0.39390 (13)	0.0256 (4)	
H11	0.4951	0.1043	0.3949	0.031*	
C12	0.3970 (3)	0.1463 (2)	0.49289 (15)	0.0367 (5)	
H12A	0.4898	0.2191	0.5325	0.055*	
H12B	0.3933	0.0580	0.5164	0.055*	
H12C	0.3044	0.1703	0.4930	0.055*	
C13	0.2577 (2)	0.0191 (2)	0.33284 (14)	0.0287 (4)	
H13A	0.2388	−0.0589	0.3684	0.034*	
H13B	0.1692	0.0557	0.3227	0.034*	
C14	0.2539 (2)	−0.0404 (2)	0.23828 (14)	0.0260 (4)	
C15	0.3865 (3)	−0.0994 (2)	0.24651 (15)	0.0309 (5)	
H15A	0.4856	−0.0254	0.2739	0.046*	
H15B	0.3793	−0.1385	0.1850	0.046*	
H15C	0.3801	−0.1722	0.2859	0.046*	
C16	0.1011 (3)	−0.1599 (2)	0.19501 (17)	0.0394 (5)	
H16A	0.0967	−0.1994	0.1343	0.059*	
H16B	0.0134	−0.1246	0.1880	0.059*	
H16C	0.0961	−0.2318	0.2352	0.059*	
C17	0.6356 (2)	0.17427 (19)	0.18083 (13)	0.0213 (4)	
C18	0.7602 (2)	0.1236 (2)	0.16206 (14)	0.0287 (4)	
H18A	0.8514	0.2045	0.1679	0.034*	
H18B	0.7930	0.0660	0.2094	0.034*	
C19	0.7075 (2)	0.0401 (2)	0.06769 (14)	0.0249 (4)	
C20	0.9213 (3)	0.1114 (3)	0.42492 (17)	0.0367 (5)	
C21	0.9193 (3)	0.2199 (3)	0.48808 (19)	0.0461 (6)	
H21A	0.9908	0.2222	0.5497	0.069*	
H21B	0.8143	0.2020	0.4909	0.069*	
H21C	0.9517	0.3092	0.4666	0.069*	

### Atomic displacement parameters (Å^2^)

**Table:**

	*U*^11^	*U*^22^	*U*^33^	*U*^12^	*U*^13^	*U*^23^
Ni1	0.01746 (13)	0.02064 (13)	0.01497 (12)	0.00797 (9)	0.00478 (9)	0.00037 (8)
Cl1	0.0266 (2)	0.0302 (3)	0.0345 (3)	0.0106 (2)	0.0132 (2)	0.0033 (2)
O1	0.0187 (6)	0.0258 (7)	0.0217 (7)	0.0075 (5)	0.0045 (5)	−0.0003 (5)
O2	0.0232 (7)	0.0264 (7)	0.0210 (7)	0.0109 (6)	0.0063 (5)	−0.0014 (5)
O3	0.0314 (8)	0.0322 (8)	0.0216 (7)	0.0082 (6)	0.0062 (6)	−0.0020 (6)
O4	0.0302 (8)	0.0473 (10)	0.0276 (8)	0.0037 (7)	0.0127 (7)	−0.0075 (7)
O5	0.0548 (12)	0.0906 (16)	0.0429 (11)	0.0162 (11)	0.0208 (9)	−0.0196 (11)
O6	0.0388 (9)	0.0625 (12)	0.0471 (10)	0.0282 (9)	0.0211 (8)	0.0056 (9)
O7	0.0582 (12)	0.0579 (12)	0.0934 (17)	0.0388 (11)	0.0410 (12)	0.0382 (12)
O9	0.0513 (12)	0.0460 (11)	0.0582 (13)	−0.0053 (9)	0.0046 (10)	0.0128 (9)
N1	0.0205 (8)	0.0244 (8)	0.0190 (8)	0.0070 (6)	0.0080 (6)	0.0001 (6)
N2	0.0221 (8)	0.0279 (8)	0.0188 (8)	0.0119 (7)	0.0050 (6)	0.0020 (6)
N3	0.0211 (8)	0.0238 (8)	0.0187 (8)	0.0086 (6)	0.0058 (6)	0.0014 (6)
N4	0.0241 (8)	0.0230 (8)	0.0185 (8)	0.0105 (6)	0.0072 (6)	0.0023 (6)
N5	0.0522 (14)	0.0499 (14)	0.0606 (16)	0.0148 (11)	0.0148 (12)	−0.0084 (12)
C1	0.0182 (9)	0.0338 (11)	0.0307 (11)	0.0060 (8)	0.0035 (8)	0.0011 (8)
C2	0.0202 (9)	0.0354 (11)	0.0242 (10)	0.0103 (8)	−0.0006 (8)	0.0013 (8)
C3	0.0288 (10)	0.0318 (11)	0.0214 (9)	0.0168 (9)	0.0089 (8)	0.0067 (8)
C4	0.0373 (12)	0.0470 (14)	0.0342 (12)	0.0252 (11)	0.0091 (10)	0.0163 (10)
C5	0.0356 (11)	0.0248 (10)	0.0271 (10)	0.0153 (9)	0.0109 (9)	0.0069 (8)
C6	0.0284 (10)	0.0222 (9)	0.0225 (10)	0.0062 (8)	0.0077 (8)	0.0034 (7)
C7	0.0290 (11)	0.0325 (11)	0.0272 (10)	0.0082 (9)	0.0126 (9)	0.0056 (8)
C8	0.0382 (12)	0.0247 (11)	0.0345 (12)	0.0025 (9)	0.0100 (10)	0.0014 (9)
C9	0.0343 (11)	0.0245 (10)	0.0195 (9)	0.0086 (8)	0.0067 (8)	−0.0026 (7)
C10	0.0331 (11)	0.0273 (10)	0.0168 (9)	0.0088 (8)	0.0050 (8)	−0.0014 (7)
C11	0.0316 (11)	0.0273 (10)	0.0200 (9)	0.0117 (8)	0.0089 (8)	0.0048 (8)
C12	0.0539 (15)	0.0362 (12)	0.0211 (10)	0.0121 (11)	0.0156 (10)	0.0071 (9)
C13	0.0329 (11)	0.0284 (10)	0.0270 (10)	0.0061 (9)	0.0163 (9)	0.0054 (8)
C14	0.0302 (11)	0.0230 (10)	0.0253 (10)	0.0064 (8)	0.0117 (8)	0.0030 (8)
C15	0.0423 (12)	0.0248 (10)	0.0320 (11)	0.0157 (9)	0.0163 (10)	0.0050 (8)
C16	0.0404 (13)	0.0302 (12)	0.0393 (13)	−0.0012 (10)	0.0132 (10)	0.0019 (9)
C17	0.0181 (9)	0.0201 (9)	0.0240 (9)	0.0043 (7)	0.0071 (7)	−0.0030 (7)
C18	0.0190 (9)	0.0415 (12)	0.0236 (10)	0.0121 (9)	0.0037 (8)	−0.0086 (8)
C19	0.0179 (9)	0.0330 (11)	0.0243 (10)	0.0129 (8)	0.0044 (8)	−0.0040 (8)
C20	0.0310 (12)	0.0379 (13)	0.0383 (13)	0.0099 (10)	0.0080 (10)	0.0062 (10)
C21	0.0508 (15)	0.0462 (15)	0.0453 (15)	0.0162 (12)	0.0213 (12)	0.0014 (11)

### Geometric parameters (Å, °)

**Table:**

Ni1—N4	2.0813 (16)	C5—H5A	0.9900
Ni1—N2	2.0868 (16)	C5—H5B	0.9900
Ni1—O2	2.1014 (13)	C6—C7	1.529 (3)
Ni1—N1	2.1144 (16)	C6—C8	1.533 (3)
Ni1—N3	2.1223 (16)	C7—H7A	0.9800
Ni1—O1	2.2603 (13)	C7—H7B	0.9800
Ni1—C17	2.5082 (18)	C7—H7C	0.9800
Cl1—O6	1.4222 (16)	C8—H8A	0.9800
Cl1—O9	1.4213 (19)	C8—H8B	0.9800
Cl1—O5	1.4306 (19)	C8—H8C	0.9800
Cl1—O7	1.4359 (19)	C9—C10	1.507 (3)
O1—C17	1.270 (2)	C9—H9A	0.9900
O2—C17	1.252 (2)	C9—H9B	0.9900
O3—C19	1.324 (3)	C10—H10A	0.9900
O3—H3o	0.83 (1)	C10—H10B	0.9900
O4—C19	1.204 (3)	C11—C13	1.526 (3)
N1—C1	1.475 (2)	C11—C12	1.535 (3)
N1—C14	1.505 (2)	C11—H11	1.0000
N1—H1	0.8800	C12—H12A	0.9800
N2—C2	1.478 (3)	C12—H12B	0.9800
N2—C3	1.491 (3)	C12—H12C	0.9800
N2—H2	0.8800	C13—C14	1.529 (3)
N3—C9	1.481 (2)	C13—H13A	0.9900
N3—C6	1.504 (2)	C13—H13B	0.9900
N3—H3	0.8800	C14—C15	1.521 (3)
N4—C10	1.478 (2)	C14—C16	1.540 (3)
N4—C11	1.491 (2)	C15—H15A	0.9800
N4—H4	0.8800	C15—H15B	0.9800
N5—C20	1.131 (3)	C15—H15C	0.9800
C1—C2	1.508 (3)	C16—H16A	0.9800
C1—H1A	0.9900	C16—H16B	0.9800
C1—H1B	0.9900	C16—H16C	0.9800
C2—H2A	0.9900	C17—C18	1.515 (3)
C2—H2B	0.9900	C18—C19	1.506 (3)
C3—C4	1.531 (3)	C18—H18A	0.9900
C3—C5	1.525 (3)	C18—H18B	0.9900
C3—H3A	1.0000	C20—C21	1.439 (3)
C4—H4A	0.9800	C21—H21A	0.9800
C4—H4B	0.9800	C21—H21B	0.9800
C4—H4C	0.9800	C21—H21C	0.9800
C5—C6	1.527 (3)		
			
N4—Ni1—N2	104.09 (6)	C5—C6—C7	111.33 (16)
N4—Ni1—O2	95.69 (6)	N3—C6—C8	111.54 (16)
N2—Ni1—O2	159.77 (6)	C5—C6—C8	108.18 (17)
N4—Ni1—N1	91.55 (6)	C7—C6—C8	108.01 (17)
N2—Ni1—N1	85.19 (6)	C6—C7—H7A	109.5
O2—Ni1—N1	98.65 (6)	C6—C7—H7B	109.5
N4—Ni1—N3	85.63 (6)	H7A—C7—H7B	109.5
N2—Ni1—N3	91.43 (6)	C6—C7—H7C	109.5
O2—Ni1—N3	85.80 (6)	H7A—C7—H7C	109.5
N1—Ni1—N3	174.96 (6)	H7B—C7—H7C	109.5
N4—Ni1—O1	154.65 (6)	C6—C8—H8A	109.5
N2—Ni1—O1	100.65 (6)	C6—C8—H8B	109.5
O2—Ni1—O1	60.22 (5)	H8A—C8—H8B	109.5
N1—Ni1—O1	85.06 (5)	C6—C8—H8C	109.5
N3—Ni1—O1	99.27 (5)	H8A—C8—H8C	109.5
N4—Ni1—C17	125.20 (6)	H8B—C8—H8C	109.5
N2—Ni1—C17	130.70 (6)	N3—C9—C10	109.34 (16)
O2—Ni1—C17	29.89 (6)	N3—C9—H9A	109.8
N1—Ni1—C17	92.09 (6)	C10—C9—H9A	109.8
N3—Ni1—C17	92.95 (6)	N3—C9—H9B	109.8
O1—Ni1—C17	30.33 (6)	C10—C9—H9B	109.8
O6—Cl1—O9	110.07 (13)	H9A—C9—H9B	108.3
O6—Cl1—O5	109.36 (12)	N4—C10—C9	109.92 (16)
O9—Cl1—O5	108.85 (13)	N4—C10—H10A	109.7
O6—Cl1—O7	110.15 (12)	C9—C10—H10A	109.7
O9—Cl1—O7	107.90 (13)	N4—C10—H10B	109.7
O5—Cl1—O7	110.50 (14)	C9—C10—H10B	109.7
C17—O1—Ni1	85.69 (11)	H10A—C10—H10B	108.2
C17—O2—Ni1	93.34 (11)	N4—C11—C13	111.13 (16)
C19—O3—H3O	110 (2)	N4—C11—C12	111.90 (16)
C1—N1—C14	113.93 (15)	C13—C11—C12	109.05 (17)
C1—N1—Ni1	104.51 (12)	N4—C11—H11	108.2
C14—N1—Ni1	120.56 (12)	C13—C11—H11	108.2
C1—N1—H1	105.6	C12—C11—H11	108.2
C14—N1—H1	105.6	C11—C12—H12A	109.5
Ni1—N1—H1	105.6	C11—C12—H12B	109.5
C2—N2—C3	112.71 (15)	H12A—C12—H12B	109.5
C2—N2—Ni1	104.89 (12)	C11—C12—H12C	109.5
C3—N2—Ni1	116.63 (12)	H12A—C12—H12C	109.5
C2—N2—H2	107.4	H12B—C12—H12C	109.5
C3—N2—H2	107.4	C14—C13—C11	119.18 (17)
Ni1—N2—H2	107.4	C14—C13—H13A	107.5
C9—N3—C6	114.03 (15)	C11—C13—H13A	107.5
C9—N3—Ni1	104.30 (11)	C14—C13—H13B	107.5
C6—N3—Ni1	120.41 (12)	C11—C13—H13B	107.5
C9—N3—H3	105.7	H13A—C13—H13B	107.0
C6—N3—H3	105.7	N1—C14—C15	107.74 (16)
Ni1—N3—H3	105.7	N1—C14—C13	110.42 (16)
C10—N4—C11	112.07 (15)	C15—C14—C13	111.55 (17)
C10—N4—Ni1	104.22 (12)	N1—C14—C16	111.13 (17)
C11—N4—Ni1	115.03 (11)	C15—C14—C16	107.64 (18)
C10—N4—H4	108.4	C13—C14—C16	108.34 (17)
C11—N4—H4	108.4	C14—C15—H15A	109.5
Ni1—N4—H4	108.4	C14—C15—H15B	109.5
N1—C1—C2	109.58 (16)	H15A—C15—H15B	109.5
N1—C1—H1A	109.8	C14—C15—H15C	109.5
C2—C1—H1A	109.8	H15A—C15—H15C	109.5
N1—C1—H1B	109.8	H15B—C15—H15C	109.5
C2—C1—H1B	109.8	C14—C16—H16A	109.5
H1A—C1—H1B	108.2	C14—C16—H16B	109.5
N2—C2—C1	109.16 (16)	H16A—C16—H16B	109.5
N2—C2—H2A	109.8	C14—C16—H16C	109.5
C1—C2—H2A	109.8	H16A—C16—H16C	109.5
N2—C2—H2B	109.8	H16B—C16—H16C	109.5
C1—C2—H2B	109.8	O2—C17—O1	120.74 (17)
H2A—C2—H2B	108.3	O2—C17—C18	118.42 (17)
N2—C3—C4	112.02 (17)	O1—C17—C18	120.80 (17)
N2—C3—C5	110.60 (16)	O2—C17—Ni1	56.76 (9)
C4—C3—C5	109.25 (17)	O1—C17—Ni1	63.98 (10)
N2—C3—H3A	108.3	C18—C17—Ni1	174.93 (14)
C4—C3—H3A	108.3	C19—C18—C17	113.04 (16)
C5—C3—H3A	108.3	C19—C18—H18A	109.0
C3—C4—H4A	109.5	C17—C18—H18A	109.0
C3—C4—H4B	109.5	C19—C18—H18B	109.0
H4A—C4—H4B	109.5	C17—C18—H18B	109.0
C3—C4—H4C	109.5	H18A—C18—H18B	107.8
H4A—C4—H4C	109.5	O4—C19—O3	123.73 (18)
H4B—C4—H4C	109.5	O4—C19—C18	124.16 (19)
C6—C5—C3	119.38 (16)	O3—C19—C18	112.10 (18)
C6—C5—H5A	107.5	N5—C20—C21	179.6 (3)
C3—C5—H5A	107.5	C20—C21—H21A	109.5
C6—C5—H5B	107.5	C20—C21—H21B	109.5
C3—C5—H5B	107.5	H21A—C21—H21B	109.5
H5A—C5—H5B	107.0	C20—C21—H21C	109.5
N3—C6—C5	110.32 (16)	H21A—C21—H21C	109.5
N3—C6—C7	107.46 (16)	H21B—C21—H21C	109.5

### Hydrogen-bond geometry (Å, °)

**Table:**

*D*—H···*A*	*D*—H	H···*A*	*D*···*A*	*D*—H···*A*
O3—H3o···O1^i^	0.83 (1)	1.84 (1)	2.669 (2)	173 (3)
N1—H1···O4^i^	0.88	2.19	3.038 (2)	161
N2—H2···O5	0.88	2.21	3.052 (2)	160
N3—H3···O6^ii^	0.88	2.44	3.291 (2)	163
N4—H4···O7	0.88	2.24	3.080 (2)	161

Symmetry codes: (i) −*x*+1, −*y*, −*z*; (ii) *x*+1, *y*, *z*.
